# Changes in Intraocular Pressure with Use of Periocular Triamcinolone Cream

**DOI:** 10.18502/jovr.v17i3.11574

**Published:** 2022-08-15

**Authors:** Diana H. Kim, Sana A. Bautista, Elana Meer, Brendan McGeehan, Maureen G. Maguire, César A. Briceño

**Affiliations:** ^1^Department of Ophthalmology, Scheie Eye Institute, University of Pennsylvania, Philadelphia, PA, USA; ^2^Perelman School of Medicine, University of Pennsylvania, Philadelphia, PA, USA; ^3^Center for Preventative Ophthalmology and Biostatistics, University of Pennsylvania, Philadelphia, PA, USA

**Keywords:** Glaucoma, Intraocular Pressure, Ocular Hypertension, Periocular Dermatitis, Steroids, Triamcinolone

## Abstract

**Purpose:**

To evaluate the effect of periocular topical triamcinolone cream on intraocular pressure.

**Methods:**

A retrospective chart review identified 57 patients, 114 eyes using triamcinolone cream (0.1%, 0.025%) with subsequent intraocular pressure (IOP) checks at three follow-up visits. Descriptive, univariate, and multivariate analyses were performed to assess effects of age, therapy duration, consecutive weeks on steroid, prescription strength, time of day, and method of measurement on IOP levels. Generalized Estimating Equations were used in regression models to account for correlation of eyes within subjects and across visits.

**Results:**

We identified 57 patients using triamcinolone cream for allergic or eczematous dermatitis of the eyelid. Prescription strengths were 0.025% or 0.1% and patients were followed for a median of 4.9 months. Measurements of IOP at baseline did not change as compared to all IOP measurements at follow-ups and did not change with steroid strength. The mean change in IOP at all follow-up visits was 0.07 mm Hg (95% confidence interval [CI]: –0.36, 0.50). After adjustment for the method of tonometer and the patient's age, the mean change was 0.03 mm Hg (95% CI: –0.68, 0.73, *P* = 0.93). Prescription strength and consecutive weeks of therapy were not associated with IOP. Two patients experienced a significant elevation in IOP of > 10 mm Hg, one through the concomitant consequences of systemic corticosteroids usage and the other through prolonged topical application.

**Conclusion:**

In patients taking periocular triamcinolone cream, there was no clinically meaningful change in mean IOP between baseline and follow-up visits, and IOP measurements were not related to variances in prescription strength or duration of therapy.

##  INTRODUCTION 

Atopic dermatitis (AD) is a common, chronic relapsing inflammatory skin condition with increasing incidence in the past decades, especially in industrialized nations.^[1]^ It affects up to 20% of children and 3% of adults worldwide^[2]^ and is most commonly treated with topical corticosteroids. It is expected that with increasing incidence of AD, including in the eyelid, the use of topical periocular corticosteroids will also increase over time.

Prolonged use of local (ocular surface, subconjunctival, sub-Tenon's, retrobulbar) and systemic corticosteroids is associated with a rise in intraocular pressure (IOP) and resultant glaucoma.^[3,4,5,6]^ Several mechanisms are thought to be responsible for this effect, including corticosteroids leading to an accumulation of polymerized glycosaminoglycans in the trabecular meshwork and increases in the trabecular meshwork's cell size and shape, leading to increased aqueous humor outflow resistance.^[7]^ Steroid potency is thought to be correlated to their ocular hypertensive effect,^[8]^ while the duration of use has also been shown to increase ocular hypertension.^[9]^ In the eyelid, increased use of topical corticosteroids may be particularly problematic given their close proximity to the eye.

Despite the growing body of literature that suggests that IOP can be elevated both in response to systemic and local corticosteroids, the effect of periocular steroids in particular is still relatively unknown. While multiple case reports have described IOP elevation or glaucoma as a direct result of periocular corticosteroid use,^[10,11,12,13,14,15]^ larger studies remain inconclusive. In a retrospective case control of 9793 patients, Garbe et al showed that nasal glucocorticoids were not associated with intraocular hypertension.^[16]^

A retrospective study performed by Tamagawa-Mineoka et al on 65 patients demonstrated no association between topical periocular corticosteroid use and IOP, whereas a study performed by Maeng et al on 31 patients demonstrated a positive correlation only in patients with baseline IOP > 14 mm Hg.^[17,18]^ The latter two studies were limited by their sample sizes, the use of multiple brands of topical corticosteroids, and differing applications of prescription strength or usage patterns.

Recognizing the limited evidence on periocular corticosteroids and IOP, the aim of this retrospective chart review was to evaluate the effect of periocular topical triamcinolone cream 0.1% and 0.025%, a commonly used topical corticosteroid in AD, on IOP. Our study seeks to identify associations with and risk factors for IOP elevation, elucidating the effect of a single corticosteroid with a defined use on IOP in real world practice.

**Table 1:** Patients' baseline characteristics.


	**Overall (** ***N*** ** = 57)**
**Age**	
Mean (SD)	56.5 (18.3)
Median (Q1, Q3)	61.0 (46.0, 68.0)
Range	19.0–88.0
**Sex**	
Female	44 (77.2%)
Male	13 (22.8%)
**Strength**	
0.025%	42 (73.7%)
0.10%	15 (26.3%)
**Duration of follow-up (months)**	
Mean (SD)	10.9 (14.8)
Median (Q1, Q3)	4.9 (2.1, 14.6)
Range	0.5–78.6
	
	
white<bcol>2</ecol>SD, standard deviation

**Table 2:** Description of IOP at each visit.


	**Baseline (** ***N*** ** = 114)**	**Visit 1 (** ***N*** ** = 114)**	**Visit 2 (** ***N*** ** = 68)**	**Visit 3 (** ***N*** ** = 42)**	**All follow-up visits (** ***N*** ** = 224)**
**IOP, mmHg**			
Mean (SD)	14.8 (2.9)	14.9 (3.6)	15.1 (3.5)	13.5 (2.9)	14.7 (3.5)
Median (Q1, Q3)	15.0 (13.0, 16.0)	14.0 (13.0, 17.0)	15.0 (12.0, 17.3)	14.0 (12.0, 15.0)	14.0 (12.0, 17.0)
Range	7.0–23.0	5.0–28.0	9.0–23.0	7.0–21.0	5.0–28.0
**Change in IOP from baseline, mmHg**					
Mean (SD)	Not applicable	0.1 (3.4)	0.5 (3.4)	–0.7 (3.0)	0.07 (3.3)
Median (Q1, Q3)	Not applicable	0.0 (–2.0, 2.0)	0.0 (-2.0, 3.0)	–1.0 (–2.8, 0.0)	0.0 (–2.0, 2.0)
Range	Not applicable	–7.0 – 16.0	–6.0 – 9.0	–6.0 – 11.0	–7.0 – 16.0
	
	
IOP, intraocular pressure; SD, standard deviation; N, number					

**Table 3:** Description of IOP by corticosteroid use and by corticosteroid strength.


	**Baseline (** ***N*** ** = 114)**	**After corticosteroid use (** ***N*** ** = 224)**		**Total (** ***N*** ** = 338)**	***P*** **-value**
**IOP, mmHg**	<@		0.81
Mean (SD)	14.8 (2.9)	<@14.7 (3.5)		14.7 (3.3)	
Median (Q1, Q3)	15.0 (13.0, 16.0)	<@14.0 (12.0, 17.0)		14.5 (12.0, 17.0)	
Range	7.0–23.0	<@5.0–28.0		5.0–28.0	
	**Baseline (** ***N*** ** = 114)**	**0.025% (** ***N*** ** = 162)**	**0.10% (** ***N*** ** = 62)**	**Total (** ***N*** ** = 338)**	***P*** **-value**
**IOP, mmHg**			0.91
Mean (SD)	14.8 (2.9)	14.6 (3.4)	14.9 (3.6)	14.7 (3.3)	
Median (Q1, Q3)	15.0 (13.0, 16.0)	14.0 (12.0, 16.0)	15.0 (13.0, 17.0)	14.5 (12.0, 17.0)	
Range	7.0–23.0	6.0–28.0	5.0–23.0	5.0–28.0	
	
	
IOP, intraocular pressure; SD, standard deviation; N, number					

**Table 4:** Univariate regression analysis of the association of features and IOP for all IOP measurements.


**Feature**	**Lower 95% confidence limit**	**Upper 95% confidence limit**	***P*** **-value**
	**Difference in IOP per unit change, mmHg**		
Age (yrs)				
	–0.04	–0.07	0.00	0.05
Consecutive weeks of usage				
	0.00	–0.09	0.10	0.93
Time of day (24-hr clock)				
	–0.17	–0.37	0.04	0.11
		
	**Model Mean IOP, mmHg**		
Sex				
Female	14.68	13.84	15.52	
Male	14.78	13.78	15.78	0.88
Strength				
None	14.76	14.05	15.47	
0.025%	14.58	13.69	15.47	0.69
0.10%	14.92	13.34	16.50	0.84
Corticosteroid Usage				
No	14.76	14.05	15.47	
Yes	14.67	13.89	15.46	0.81
Method of Measurement				
Applanation	14.21	13.36	15.06	
Other	15.14	14.24	16.04	0.11
	
	
IOP, intraocular pressure				

**Table 5:** Results of regression analysis for IOP with corticosteroid use as covariate of interest.


**Term**	**Estimate**	**Lower 95% CI**	**Upper 95% CI**	** P -value**
Intercept	16.38	14.06	18.71	< 0.001
Corticosteroid-Yes	0.03	–0.68	0.73	0.928
Age	–0.04	–0.07	0.00	0.025
Non-applanation measurement method	1.07	0.00	2.13	0.049
	
	
CI, confidence interval				

**Table 6:** Results of regression analysis for IOP with corticosteroid strength as covariate of interest.


**Term**	**Estimate**	**Lower 95% CI**	**Upper 95% CI**	** P -value**
Intercept	16.43	14.11	18.75	< 0.001
Strength 0.025%	0.08	–0.82	0.98	0.859
Strength 0.10%	–0.09	–1.55	1.37	0.908
Age	–0.04	–0.07	0.00	0.025
Non-applanation measurement method	1.07	0.00	2.14	0.050
	
	

##  METHODS 

A chart review was performed of all patients at the University of Pennsylvania with AD of the eyelids treated with topical periocular triamcinolone creams 0.1% and 0.025% twice daily from January 2010 to February 2020. Patients were identified through billing codes for allergic dermatitis of the eyelid and eczematous dermatitis of the eyelid. Patients with a diagnosis of glaucoma or ocular hypertension prior to the initiation of treatment were not included. Patients who were included were required to have a baseline IOP prior to the initiation of treatment and at least one IOP measurement after the initiation of treatment. Demographic data including age, sex, triamcinolone dose, IOP measurements, method of measurements, and time of measurements were recorded for baseline (pretreatment) and at follow-up visits for those patients who remained on the corticosteroid treatment. Univariate and multivariable regressions were performed using the aforementioned covariates. In the multivariable settings, corticosteroid use and prescription strength were the covariates of interest in two separate models. Additional covariates were utilized in a backward selection procedure based on a *P*-value cutoff of *P* ≤ 0.05 for model inclusion. Generalized estimating equation regression (GEE regression) was used to analyze changes between baseline IOP and IOP at follow-up visits to account for inter-eye correlation and repeated measures. All analyses were performed using R Version 3.5.1. This study was determined to be exempt from review by the University of Pennsylvania institutional review board. This study complies with the tenets of the Declaration of Helsinki and Health Insurance Portability and Accountability Act regulations.

##  RESULTS

Fifty-seven patients were identified as having had both a baseline visit and at least one subsequent follow-up visit at which they were still on triamcinolone. The mean age was 56.5 years (18.3%) and 44 (77.2%) were female (77.2%) [Table 1]. Triamcinolone cream prescription strengths were 0.025% in 42 patients (73.7%) and 0.10% in 15 patients (26.3%). Overall mean duration between baseline and any follow-up visit was approximately 10.9 months, with a median of 4.9 months.

Baseline analysis of the 114 eyes of 57 patients revealed a mean IOP of 14.8 ± 2.9 mm Hg (*n* = 114). A total of 57 patients (*n* = 114) were available for an initial follow-up (visit 1), 34 patients (*n* = 68) were available for a second follow-up (visit 2), and 21 patients (*n* = 42) were available for a third follow-up (visit 3). Mean IOPs for visit 1, visit 2, and visit 3 were 14.9 ± 3.6 mm Hg, 15.1 ± 3.5 mm Hg, and 13.5 ± 2.9 mm Hg, respectively [Table 2]. The average time from baseline to visit 1, visit 2, and visit 3 was approximately 4.3 months, 9.4 months, and 17.7 months, respectively [Supplemental Table 1].

Measurements of IOP at baseline (*n* = 114, 14.8 mm Hg, 95% CI: 14.1–15.5) did not change as compared to all IOPs at follow-ups (*n* = 224, 14.7 mm Hg, 95% CI: 13.9–15.5, *P* = 0.81). Estimated mean change was –0.1 mm Hg (95% CI: –0.8 to 0.6). Further, the mean differences in IOP from baseline to visit 1, visit 2, and visit 3 were 0.1 ± 3.4 mm Hg, 0.5 ± 3.4 mm Hg, and –0.7 ± 3.0 mm Hg, respectively. Measurements of IOP at baseline (*n* = 114, 14.8 mm Hg, 95% CI: 14.1–15.5) also did not change at follow-ups based on triamcinolone strengths of 0.025% (*n* = 162, 14.58 mm Hg, 95% CI: 13.7–15.5, *P* = 0.69) or 0.1% (*n* = 62, 14.92 mm Hg, 95% CI: 13.3–16.5, *P* = 0.84). Estimated mean difference for 0.025% was –0.2 mm Hg (95% CI: –1.1 to 0.7) and for 0.1% was 0.1 mm Hg (95% CI: –1.3 to 1.6) [Table 3]. Only two patients (3.5%) and three eyes (2.6%) had a clinically meaningful IOP change by 10 mm Hg or more at any time point. One of the two patients who experienced this IOP change was on concomitant maintenance oral prednisone for immune thrombocytopenia and exhibited this IOP increase after 1.3 months of topical triamcinolone use. The second patient experienced IOP elevation at 19 months of continued triamcinolone use. He was unable to be located for follow-up and remained on triamcinolone longer than was recommended.

The distribution of time and method of measurement (applanation, Icare, Tono-pen) was similar for baseline and follow-up visits [Supplemental Table 2]. Concordance in the method of IOP measurement between baseline and subsequent visits was also described. At any visit, at least 50% of eyes were measured with the same method of IOP measurement that was used for the baseline visit. From baseline to all visits, 25 (43.9%) patients had IOP measured by the same method of tonometer [Supplemental Table 3].

In univariate analysis, a –0.04 mm Hg in IOP (95% CI: –0.7 to 0.00; *P* = 0.05) was estimated for every year of age. Other variables including consecutive weeks of usage, time of day, sex, triamcinolone usage, triamcinolone strength, and method of measurement were not significantly associated with IOP [Table 4].

After backward variable selection, only age and the non-applanation method of measurement were retained as statistically significant features associated with IOP. When IOP changes from baseline were estimated for corticosteroid use and adjusted for these two factors, the mean change was 0.03 mm Hg (95% CI: –0.68 to 0.73; *P* = 0.928) [Table 5 & 6]. When IOP change from baseline was estimated for each of the two concentrations of triamcinolone cream and adjusted for age and non-applanation method of measurement, the mean change was 0.08 mm Hg (95% CI: –0.82 to 0.98; *P* = 0.86) for 0.025% concentration and –0.09 mm Hg (95% CI: –1.55 to 1.37; *P* = 0.908) for 0.10% concentration [Tables 5 & 6]. Older patients had lower IOP by –0.04 mm Hg per year (95% CI: –0.7 to 0.00; *P* = 0.025) in both models. Non-applanation measurement methods increased IOP by 1.07 mm Hg (95% CI: 0.00–2.14, *P*=0.050) in both models.

##  DISCUSSION

Our retrospective dataset demonstrates that the mean IOP change in eyes of patients after use of triamcinolone cream 0.1% or 0.025% is near zero in those who have subsequent IOP measurements at follow-ups. Neither dose nor consecutive weeks of therapy were associated with IOP increases, suggesting that cumulative dosing does not have an effect. In our multivariate analysis, only age and method of measurement correlated with changes in IOP at follow-up visits. Given that our patients were on average 56 years of age, the decrease of 0.04 mm Hg in IOP per one year of age is consistent with previous reports that IOP decreases with age beyond the sixth decade.^[19,20]^ Our finding that non-applanation measurements with either Tono-pen or ICare elevate IOP is also consistent with the literature.^[21,22]^

Topical periocular corticosteroids are currently the treatment of choice for periocular inflammatory conditions such as AD. There is currently no consensus on the risk of IOP elevations from periocular corticosteroid use, which may create uncertainty in providers who wish to initiate therapy. Our study is reassuring in that it suggests that triamcinolone cream 0.1% and 0.025% may be safe over several months of corticosteroid use without statistically significant elevations in IOP. Taken together, our study corroborates previous reports that topical triamcinolone or topical corticosteroids are not associated with changes in IOP.^[17,23]^ Our results may help guide management for medical providers who may be reluctant to use corticosteroids, preferring alternatives such as calcineurin inhibitors which are associated with increased risk of other adverse effects such as local skin irritation, burning, and higher costs.^[24]^

Despite the results of our study, we identified two patients (one of whom was already taking concomitant oral steroids and another was on triamcinolone longer than was intended) with IOP elevations that may be connected to using topical triamcinolone cream. While a causal relationship cannot be established, it follows that elevation in IOP after triamcinolone use is certainly possible and further studies need to address exactly which patient populations are at highest risk. Given the limited number of patients with an increase in IOP after triamcinolone use, no subgroup analysis could be performed. Thus, providers should still recognize that the risk of IOP elevation from periocular triamcinolone still exists in those with concomitant systemic steroid or excessive, prolonged use.

Our study appears to be the largest analyses to date in assessing associations between a single corticosteroid used for a single diagnosis and IOP outcomes. Moreover, we study a patient population who receive corticosteroids in a single practice, reflecting the heterogeneous adherence to steroid applications and their intervals of follow-up.

Our study is limited by its retrospective nature and relatively small cohort of patients. IOP outcomes are inherently influenced by diurnal circulation, method of tonometry, and style of examiner. We nevertheless showed that the time of the day and the method of tonometry did not differ [Supplemental Table 2], and that patients consistently received the same type of repeated IOP measurement in > 50% of visits [Supplemental Table 3]. Our study was also limited by the single IOP measurement done only at each visit. Further evaluation and analysis of these variables that influence the outcomes of IOP measurements in addition to the execution of repeat measurements at follow-up visits may help control for such variances.

As is typical in real world practices, the follow-up times in our study varied and resulted in a wide range of times between visits throughout the sample. The duration of follow-up when including all three visits from baseline was approximately an average of 10 months, while the duration from baseline to the first visit was an average of 4 months. While some patients had shorter follow-ups, our follow-up duration captured the prolonged window of time in which IOP elevations can develop after steroid use, which is typically about one month.^[3,7,25]^ There is also evidence that IOP elevation may occur immediately after initiation of therapy as there is evidence that IOP elevation may occur early if at all after intravitreal triamcinolone injections.^[26]^ However, patients with shorter follow-ups did not exhibit differences in IOP as compared to those with longer ones. Patients with previous IOP elevations from known corticosteroid use or those suffering with glaucoma were also excluded from our analysis.^[7,17]^ Unfortunately, family history was not recorded in our review so it is possible that patients with undiagnosed glaucoma were included in our study. Two patients in our evaluation as described above had clinically significant IOP elevations. Patients whose initial measurement of IOP was > 20 mmHg in either eye did not have clinically significant elevations in IOP in our study. Case reports also suggest that IOP elevations may be more dramatic in younger patients, which is also reflected in follow-up measurements over many years.^[10,13,14]^ Our study patient population is much older. Finally, as all corticosteroids are unequal in their properties, it is possible that corticosteroids other than triamcinolone that were not studied may be associated with IOP elevations. Triamcinolone, like many other topical corticosteroids, are prescribed often as needed and used with inconsistency that may confound results. In our study, the vast majority of patients were prescribed triamcinolone twice daily or to be used as needed.

In summary, in our retrospective analysis of 114 eyes, we demonstrated that IOP measurements do not change after the initiation of periocular triamcinolone cream, but varied ages and methods of measurement influence IOP. During our evaluation an anomaly was discovered where two patients experienced clinically significant IOP elevation, one through the concomitant consequences of systemic steroid use and the other from the excessive prolonged topical usage for 19 months, suggesting that these may serve as risk factors for elevated IOP. It is recommended that patients with either systemic steroid use or excessively prolonged topical usage be more closely monitored. In our study, however, our results serve to reassure providers that topical triamcinolone can be safely prescribed without a significant effect on IOP.

##  Ethics Approval

This study was determined to be exempt from review by the University of Pennsylvania institutional review board. This study complies with the tenets of the Declaration of Helsinki and Health Insurance Portability and Accountability Act regulations.

##  Financial Support and Sponsorship

Nil.

##  Conflicts of interest

The authors declare that they have no conflict of interest.
